# Accommodating to motor difficulties and communication impairments in people with autism: the MORE intervention model

**DOI:** 10.3389/fnint.2013.00045

**Published:** 2013-06-18

**Authors:** Anne Emerson, Jackie Dearden

**Affiliations:** School of Education, University of NottinghamNottingham, UK

**Keywords:** autism, motor impairment, severe communication impairment, expectations, literacy

## Abstract

Motor impairment in individuals with autism potentially impacts on their development in all spheres. This paper is particularly concerned with people with severe communication impairments suggesting that recognition of the impact of motor impairments on their lives could lead to more effective interventions being developed. One such intervention is the MORE (Means, Opportunities, Reasons, and Expectations) model, founded on the “least dangerous assumption,” that is assuming competence until otherwise established through long-term observation and assessment. Components of the model include recognizing the importance of having high expectations and linking this to the way people are spoken to; timing within an intervention and over long periods; the importance of eye-hand coordination and teaching independent pointing skills. It is suggested that literacy should be offered as an early step which could significantly enhance communication.

## Introduction

There is increasingly widespread recognition of the relevance of motor impairments to the lives of people with autism (Boucher, 2003; Ming et al., 2007; Hilton et al., 2012; Liu, 2012). These impairments are thought to be present from birth and potentially the earliest diagnostic markers of autism (Mitchell et al., 2006; Iverson and Wozniak, 2007). It is also suggested that motor impairment may be a core deficit in autism (Dziuk et al., 2007). Researchers have begun to consider the link between ability, as measured by I.Q., and the presence, to varying degrees, of motor impairments (Mari et al., 2003) as well as the link between sensory-motor difficulties and the development of communication (Iverson and Wozniak, 2007). Motor impairments have so far mostly been considered in terms of their recognition and diagnosis but are also of considerable relevance to intervention, at all stages of development. This paper suggests a model to aid understanding of people with autism and severe communication impairments, in the light of possible motor difficulties, and offers suggestions for interventions. The term “motor” is used to suggest a wide range of skills and actions, with “movement” denoting a specific function comprising a range of motor skills.

## The impact on development

Studies of the motor skills of people with autism sit within the wider fields of perceptual and sensory differences (Minshew et al., 1997; Milne et al., 2002; Zwaigenbaum et al., 2005). Motor impairments in a baby will influence their development: “when motor development is delayed, opportunities to engage with and learn about the environment and social partners in new and different ways may be limited or hampered” (Iverson and Wozniak, 2007, p. 166). Early vocalizations and accompanying movements are entwined in terms of development (Iverson and Wozniak, 2007). Sensory-motor difficulties are likely to inhibit or prevent the development of speech communication, but due to difficulties in performing basic motor skills (Mari et al., 2003) are also likely to impact on non-verbal and augmentative and alternative communication (AAC) approaches (Mirenda, 2003a). A link between autism and effective completion of motor tasks, both when imitating and to verbal command, has been established (Haswell et al., 2009) and in both personal accounts (Chamak et al., 2008) and research (Chen et al., 2012) there is increasing emphasis and awareness of the importance of understanding the process of executing an action.

Comorbidity with other developmental disorders (Green et al., 2002; Wetherby et al., 2004) makes it difficult to ascertain whether motor impairments are specific aspects of autism or rather relate to cognitive impairment and communication difficulties. The direction of causation is not yet clear, i.e., whether motor difficulties are an aspect of cognitive impairments, or conversely whether being born with a motor impairment, particularly when it is not recognized as such, inhibits the development of cognitive and communication skills.

## Physical support for pointing

Awareness of difficulties in motor planning and execution in children and adults with autism and the potential benefits of teaching pointing were highlighted through the Facilitated Communication (FC) controversy (Biklen and Cardinal, 1997; Mostert, 2001). In this technique physical support for pointing is provided by a facilitator, which makes the origins of any resulting communication unclear. Most FC research has suggested that facilitators inadvertently influence the communication partner's pointing although there is also evidence that some individuals find FC beneficial (Emerson et al., 2001; Zanobini and Scopesi, 2001; Tuzzi, 2009; Grayson et al., 2012). The use of FC is problematic, not just because of doubts about the origins of any ensuing communication but also due to the extent to which the technique builds dependence on the facilitator rather than independence. Although people are reported to have reached independence through intensive practice with gradually faded physical support (Beukelman and Mirenda, 1998; Broderick and Kasa-Hendrickson, 2001) many FC users remain reliant on the facilitator to produce coherent communication. However, the physical support aspect of FC may not be necessary to teach pointing, and could be avoided. It is contested here that many individuals can be helped toward better communication through aspects of the original approach of FC, without physically facilitating their pointing but rather by specifically teaching pointing at an early age.

## The least dangerous assumption

Interventions for people with motor impairments can be guided by the principle of the “least dangerous assumption” (Donnellan, 1984). To illustrate, if a verbal instruction is not responded to, rather than coming to any conclusions about a person's level of understanding or willingness to conform, many possible explanations for the lack of response are systematically tested through a “trial and error” approach. Underlying this is the belief that it is possible for a person who outwardly has few independent skills to have understanding of language, knowledge, and even literacy skills that they are not able to independently demonstrate. Difficulties in the realm of executive function (Grayson, 1997) or other motor difficulties (Leary and Hill, 1996) may prevent demonstration of ability. A case study of Jack (Emerson and Dearden, 2013) is a good example of this. Ten year old Jack had very limited communication despite years of education and provision of AAC means such as signs and symbols. He was thought to have limited comprehension of speech, based on his poor level of response and his obsessive and ritualized behavior. Intervention, which at no point utilized any physical support, demonstrated that given structure Jack could independently point to pictures and words to answer increasingly complex questions and to start to express his needs and preferences. He demonstrated much higher verbal comprehension than his school had expected, a rapid rate of learning new tasks, and literacy skills (reading single words and short phrases) that had not been taught.

Part of applying the “least dangerous assumption” therefore is to have high expectations. In practice this means suspending judgments based on appearance and the initial responses of an individual and continuous long-term assessment through intervention. This starts from observations of a person's motor skills, both when they are engaged and not engaged in activities, interacting with others or alone. Abilities in hand use or coordination may be demonstrated in one task, but not in another, for example when given an instruction. The ensuing investigation considers what is needed for the task to be accomplished successfully (Wood et al., 1976; Vygotsky, 1978). This usually involves “experimenting” with more challenging and interesting activities whilst considering the need to scaffold the communication element.

## The MORE model

The challenge of working with children and adults with autism and severe communication impairment has resulted in the development of a model of intervention named MORE (Means, Opportunities, Reasons, and Expectations), based on the earlier Means, Reasons, and Opportunities developed by Money and Thurman (1994). The MORE model has been developed in relation to people who have no effective speech or alternative communication, with the objective of helping them to learn to point independently, to engage with other people and indicate their needs. The ultimate aim is for literacy to be used for communication where possible, through either pointing to whole words or spelling, to give maximum freedom of expression. The short-term aim is to find a variety of ways people can respond through pointing, to join in an interaction and increase their level of sociability and general responsiveness and therefore begin to demonstrate their understanding, knowledge and interests. What follows is a perspective on best practice with children with autism and severe communication impairment.

Focus on motor difficulties is set within an understanding of a disabled individual's dependence on context, relationship and environment in the MORE model. The first element of the model, “means,” relates specifically to ways in which someone does or might communicate e.g., use of their hands, eye-pointing, or vocalization. “Opportunities” refers to the varying situations that someone experiences and the ways in which these facilitate or impede communication (Sigafoos, 1999). Opportunities also relate to extrinsic motivation, provided by people in, or aspects of, the environment (Sigafoos et al., 1994). Carers need to be aware and vigilant of the impact of their actions on people's communication. An individual's intrinsic motivation to express themselves is termed “reasons,” it is evidently difficult to influence this at times, and it is the responsibility of educators to recognize what motivation someone may have and to keep investigating until they have found something that might result in an effort to communicate. “Expectations,” in the model, as already expressed, are the key to persistence and fundamental in not limiting what someone might achieve (Mirenda, 2003b; Udistsky and Hughson, 2012).

The MORE approach has important components as described below:

- **Timing (within an interaction)**. Either waiting to respond or responding at an appropriate time pose difficulties for many people with autism (Akmanoglu-Uludag and Batu, 2005). In speech silent pauses are usually filled after about 1 second although communication partners will generally accommodate to a speaker who they perceive to be searching for words (Higginbotham and Wilkins, 1999). For alternative communication system users “failing to negotiate an alternate time order means that the very same person may, in another context, be construed as a difficult, suspect and communicatively incompetent individual” (Higginbotham and Wilkins, 1999, p. 77). In MORE interventions practitioners ask something once, in carefully articulated and phrased language, and then wait, possibly up to a minute, before prompting. This can lead to responses that would either not have been elicited or may have appeared to have been inappropriate if a different instruction had been moved on to. This may be due to a person's long linguistic processing time or to executive function impairment leading to difficulties in organizing and executing a response. It is also possible that a lack of response has become a habitual state, as a form of learned helplessness (Peterson et al., 1993).
- **Timing (across months/years)**. The rapidity of the reported progress in communication development made by people with autism and severe communication impairments when using FC were one of the aspects that added to controversy about the technique. When working on independent communication progress tends to be slow. In the case study described above (Emerson and Dearden, 2013) Jack made considerable progress in the first 4 months, as he demonstrated within that time that he could point to pictures and to words in answer to questions. More typically people make slower progress, and part of the philosophy of high expectations is to continue with intervention despite the absence of response. This obviously has resource issues, and means ensuring that teachers and parents who are with the child all the time adopt the intervention model. A case study of two children (Dearden and Emerson, in preparation) describes how one moved from minimal response to an adult, to pointing independently to pictures in a book while making full eye-contact over a period of 3 years. At one level this was minimal progress, another view is that despite already being 10 years old at the start of the intervention by the age of 13 he had a better foundation for further learning and development as a communicator.
- **Awareness of motor difficulties**. The way in which tasks are scaffolded appears to be key, particularly the need to separate the cognitive load from the motor and provide specific support to each aspect. Using pointing for a wide range of tasks starts this process, as the point usually takes the place of a more complex motor action such as speaking a word or making a gesture. It does, of course, mean that the person doing the pointing is reliant on what he or she is given to point at. To encourage engagement a child who did not appear to have any functional use of his hands was encouraged to complete a jigsaw, by eye-pointing to one of just two pieces removed from the completed puzzle. This was then gradually increased to a larger number of missing pieces. In another example (Emerson and Dearden, 2013), in order to assess Jack's understanding of a story he had been read he was given speech bubbles with phrases relating to what particular characters had said. For example “you look silly” written in a speech bubble, required a point to a picture of the person in the story who said this. Both of these activities could be accomplished by finger or eye-pointing to separate the cognitive from the motor functions.
- **Teaching pointing**. When someone with autism has no apparent ability to point accurately there may be a role for hand-over-hand guidance to establish the correct motor pattern or alternative method of training specific movements (Patton and Mussa-Ivaldi, 2004) with these techniques used alongside independent pointing practice. For the latter an emphasis needs to be placed on finding resources that entice someone to touch or manipulate. Once someone is motivated to engage with a resource it is much easier to mould their movement into a more functional and purposeful pattern. An example of this was a child who during most interactions with adults screamed and banged her head on the wall. After much trial and error it was discovered that she was motivated to open tiny flaps in a book and for the first time would bring her hand to the page. She first received help to lift the flaps but soon learned to do it herself which resulted in her behavior calming and engagement in the task.
- **Importance of teaching eye-hand coordination**. Since many children with autism do not coordinate their hand movements with eye-gaze (Dawson and Watling, 2000) part of teaching motor skills is to encourage clear looking prior to moving, to increase accuracy. Eye-hand coordination often appears to break down at the planning level of movement (Johansson et al., 2001). Impulsive movements may govern hand use prior to the person processing an instruction and looking for the target. Successful responses can sometimes be increased through a structure of gently holding the person's hands still and telling them to wait while they listen, think and look. Once they have been seen to look the gentle hold is released in order for them to respond.
- **Importance of literacy**. As will be evident the MORE model does not follow a developmental approach to children with autism. In relation to literacy this means that there are no pre-requisites before offering the opportunity to respond to written words. “A major discovery of recent literacy research is that children construct ideas about writing and written language as they do in other symbolic systems well-before they receive formal instruction in that domain, and they proceed to construct knowledge throughout the learning process” (Ravid and Tolchinsky, 2002, p. 421). Experience has shown that many people with autism and severe communication impairment, whether they have had access to formal literacy teaching or not, demonstrate recognition of at least some words. It also appears that written words are motivating, perhaps as a novel tool to be included in an intervention, or because they offer the individual an opportunity to demonstrate skill and knowledge they cannot otherwise do. Once Jack had been demonstrating literacy skills in MORE interventions he spontaneously turned to words to make demands of school staff. This included scanning pages of text for the word “computer” and taking the document to a staff member while pointing at the word.
  Most practitioners use symbols and pictures with people who have severely impaired communication (Mirenda, 2003a). They are also used in MORE, but always in conjunction with and second to, the written word, until it is clear that someone cannot learn to read. One reason for this is if the person is required to learn a new language in terms of a set of symbols, it would be better to focus their efforts on learning a much more accepted and widely used communication system such as written words.
- **Use of “full” language**. Advice regarding good practice when talking to people with autism is to use restricted language comprising single words or very short phrases (Potter and Whittaker, 2001). However, there is a risk, if restricted language is used from the beginning, that people will not have the opportunity to demonstrate a greater capacity for understanding (Emerson and Dearden, 2013). The use of restricted language also removes the good language model from which they might learn. In the MORE model the suggestion is that “full” language is used, with long pauses for processing if necessary, with visual and gestural support to aid understanding.
- **Expectations**. Finally, as already mentioned, expectations are one of the most powerful factors in performance (Rist, 2000). This has been little considered in relation to people with autism and severe communication impairments. The expectations we have of someone determines the opportunities we give them (Dale et al., 2006). It is possible that the “untapped potential” of people with autism and severe communication impairment remains hidden as a result of their considerable motor difficulties, in terms of initiating, coordinating and executing tasks, leaving them almost entirely dependent on others.

## Adoption of the MORE model of intervention

The MORE model of intervention needs evaluation to measure its effectiveness, however, even if this can be demonstrated its' usefulness will depend on people who are permanently involved in the life of the disabled person being convinced of its potential and trained in its adoption. Educators generally need to see the level of progress possible in someone they know before they will accept the power of higher expectations. The slow rate of progress, and the lack of belief that the intervention will have an effect, means that most people do not persist for long enough, and even if they want to continue resources may prevent them.

In conclusion it is argued that pointing can be enormously empowering and must be overtly taught to all children through carefully scaffolded tasks and activities. The “least dangerous assumption” should be adopted for all children in terms of their level of understanding and cognitive ability. Educators need to operate from a belief in capacity and ability, not disability, until many years of individually designed interventions, not based on the developmental model, have been investigated.

### Conflict of interest statement

The authors declare that the research was conducted in the absence of any commercial or financial relationships that could be construed as a potential conflict of interest.
